# Repetitive Transcranial Magnetic Stimulation in Post-stroke Motor Recovery: A Narrative Review

**DOI:** 10.7759/cureus.100791

**Published:** 2026-01-04

**Authors:** Jordan Stewart, Karim Alabbas

**Affiliations:** 1 General Surgery, Cardiff University, Cardiff, GBR; 2 General Medicine, University Hospital of Wales, Cardiff, GBR

**Keywords:** physical medicine and rehabilitation, recurrent transcranial magnetic stimulation (rtms), repetitive transcranial magnetic stimulation (rtms), stroke, transcranial brain stimulation, transcranial magnetic stimulation (tms)

## Abstract

Transcranial magnetic stimulation (TMS) can be used as an additional treatment for stroke recovery. Stroke is a major cause of disability in the UK, often leaving survivors with ongoing motor problems. There are different theories on motor recovery after stroke, with some suggesting that undamaged pathways in the brain help compensate for the damage, while others propose that the healthy side of the brain takes over the lost functions. Additionally, there is a model suggesting that the brain's hemispheres compete, with the healthy side inhibiting the affected side. Repetitive transcranial magnetic stimulation (rTMS) uses electromagnetic pulses to stimulate specific areas of the brain, aiming to enhance new pathways and improve patients' ability to move. Studies have shown that rTMS can be effective and safe in stroke rehabilitation, leading to better motor performance. Integrating rTMS into standard stroke rehabilitation programs could have significant benefits; however, more research is needed to determine the best treatment parameters and identify which patients could benefit the most. It is also important to combine rTMS with other therapies, such as physical and occupational therapy, for a comprehensive approach. This review highlights the potential of rTMS as an additional treatment for stroke recovery. By stimulating the brain, rTMS may help improve motor function and enhance rehabilitation outcomes. Further studies are necessary to optimize its use and determine its effectiveness in different stroke patient groups.

## Introduction and background

In the United Kingdom, stroke is the leading cause of severe disability. Current estimates suggest that over 100,000 new or recurrent cases of stroke occur in the UK each year. Of those who survive, two‑thirds find themselves living with a new disability. Significant progress throughout the past decades has been made in acute stroke management, subsequently leading to an overall reduction in mortality, and more people requiring neurorehabilitation services [1]. Prior to modern developments in neuroplasticity research, it was believed that in adulthood, the organisation of the central nervous system maintained a static nature. Therefore, any insult to its parenchyma would be irreversible, as no mechanism exists to replace the affected neurons. However, in practice, varying degrees of motor recovery are observed [2,3]. Our understanding of how motor function may be restored is limited, and when coupled with financial pressures and time constraints, the focus of neurorehabilitation shifts towards using compensatory techniques to maintain functionality, rather than reducing neurological impairment. This approach may promote learned non‑use of affected limbs, consequently leading to persistent functional limitations.

Stroke is a common, acute neurovascular disorder that produces neurological deficits, the most common of which is varying degrees of motor impairment. This arises due to the impedance of blood flow to neurological structures, either through ischaemic occlusion or haemorrhage of the arteries supplying part of the brain [3]. Cerebral tissue injury often precipitates significant electrophysiological and metabolic changes within the neural networks of the affected area. However, beyond the affected area, there is also a change in the balance between inhibitory and excitatory activity both in the affected and contralesional hemispheres, as well as in subcortical and spinal regions [4]. Theoretical models have been proposed to explain this adaptive response to cerebral damage, one of which is the bimodal balance recovery model, which introduces the concept of a structural reserve - referring to how undamaged neural pathways contribute to the recovery of motor function. The vicariation model suggests that the activity of the contralesional hemisphere and perilesional cortex replaces the lost functions of the affected areas. In contrast, the interhemispheric competition model focuses on mutual suppression between the cerebral hemispheres; it proposes that the contralateral hemisphere suppresses motor signals of the ipsilateral hemisphere, such as to reduce interference to the ipsilateral descending motor tracts. Therefore, an insult to the cerebrum in one hemisphere would, theoretically, disrupt the motor suppression from the affected hemisphere, reducing inhibition to the contralesional side [5]. Both models can be used as predictors of motor recovery; however, in the presence of greater structural reserve, the interhemispheric competition model better predicts recovery than the vicariation model, and with lower structural reserve, the opposite is true. These models form the rationale behind the application of non‑invasive brain stimulation techniques as an adjuvant neurorehabilitation intervention, particularly the use of repetitive transcranial magnetic stimulation (rTMS). The National Institute for Health and Care Excellence (NICE) does not currently recommend transcranial magnetic stimulation (TMS/rTMS) for post-stroke motor recovery primarily because the evidence is insufficient to demonstrate meaningful, sustained functional benefit - primarily guided by the Cochrane review evidence base, which shows that although rTMS can produce short-term improvements in motor impairment measures (e.g. Fugl-Meyer scores or gait parameters), these effects are small, heterogeneous, and inconsistently translate into improvements in activities of daily living (such as Barthel Index scores). This article explores the current narrative surrounding the widening application of rTMS as an adjunct for stroke recovery.

## Review

Search criteria

The literature for this narrative review was identified primarily through PubMed, Google Scholar and Scopus, which were selected due to its comprehensive coverage of peer-reviewed biomedical and neuroscience research and its suitability for identifying both mechanistic and clinical studies relevant to rTMS and stroke rehabilitation; searches combined terms related to stroke, repetitive transcranial magnetic stimulation, motor recovery, neuroplasticity, and interhemispheric inhibition, with additional landmark and clinically relevant studies identified through reference list screening of key reviews and trials, while prioritising systematic reviews and randomised or sham-controlled studies reporting validated motor or functional outcomes and clearly defined stimulation protocols, and deprioritising case reports or non-stroke populations due to limited generalisability and higher risk of bias.

Narrative review

Initial improvements in both consciousness and motor function following a stroke can be attributed to the resolution of massive cerebral oedema, as well as reperfusion of the ischaemic tissue by way of collateral arterial supply [2]. Following these processes, much of stroke recovery is posited to be a result of neurological mechanisms, primarily the recruitment of homologous pathways and re‑utilisation of redundant neuronal connections surrounding the ischaemic area. This reorganisation permits the formation of new neural networks to take over the function of areas affected by stroke. Biochemical changes within the ischaemic tissue may also assist in the preservation of functional reserve, as well as induce a regenerative response to cerebral injury through neuronal growth responses, yielding a new neural network with altered and compensatory connectivity patterns [6]. Current neurorehabilitation practice emphasises impairment reduction and true recovery through high-intensity, task-specific training. Understanding the ways in which functional deficits may be reversed through physiological means and how the processes may be supported and accelerated is vital for the progression of neurorehabilitation research, which in turn could produce more favourable patient outcomes. 

Understanding the mechanisms that permit or impede post‑injury plasticity in the cortex is crucial to the development of rehabilitative methods involving direct stimulation of the cortex. Pino et al. proposed that two possible mechanisms could influence the recovery of motor function: the interhemispheric interference imbalance and compensatory vicariation. According to this theory, the mechanism influencing recovery would be determined by the degree of damage to the cortex, or, namely, the 'structural reserve' present - a concept named the bimodal balance recovery model [5]. Vicariation is a theory that has long been used to explain regained neurological function following direct injury to the cortex. This theory proposed that the cortex may reorganise such that other unrelated areas of the cortex can overtake functions that they had not performed prior. It is thought that vicariation is employed where there is extensive damage to the primary motor cortex, as well as other areas of the motor cortex. In these cases, where there is a lower structural reserve, increased excitability in the contralesional motor cortex, detected by functional magnetic resonance imaging (fMRI), is conducive to better motor recovery. Therefore, the application of rTMS in these cases focuses on reinforcing this signalling, thereby increasing the rate at which positive neuroplastic changes may occur, while also strengthening new neural networks.

**Figure 1:**
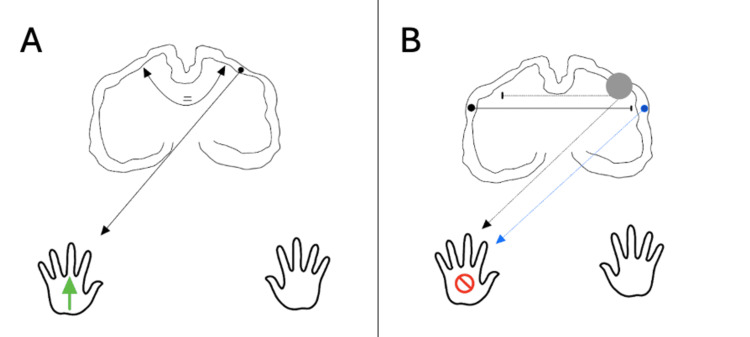
Normal and abnormal interhemispheric inhibition A) Normal interhemispheric inhibition: In a healthy brain, interhemispheric inhibition is balanced (represented by the double-ended arrow). Motor input from the ipsilateral side of motor output is inhibited during movement. B) Pathological Interhemispheric Inhibition: The cortical lesion (grey) damages inhibitory neurons in the affected hemisphere that normally control transcallosal inhibition. This means inhibition from the healthy hemisphere is no longer balanced and becomes excessive, suppressing motor signals from surviving pathways on the affected side. As a result, perilesional plasticity (blue arrow) is reduced, limiting neurological recovery. Image credit: authors Stewart J, Alabbas K

The classic theory guiding methods of non‑invasive brain stimulation is interhemispheric inhibition (Figure 1). Changes in cerebral excitability following a stroke can affect the balance of signalling between the two cerebral hemispheres, which is vital for the execution of bimanual and unilateral motor function. Interhemispheric inhibition is the mechanism through which one hemisphere inhibits the other via the corpus callosum during unilateral motor output, thereby preventing mirrored movements in the passive limb. It is thought that this process is mediated by transcallosal inhibitory glutamatergic neurons, which act directly on interneurons located in the contralateral motor cortex. Therefore, the damaged hemisphere is unable to counteract the inhibitory signalling originating from the opposing cortex, allowing it to freely impede motor signalling from surviving regions of the cortex. In such cases, it is posited that the structural reserve is greater, and the damaged area may be limited to only part of the motor cortex. Increased signal intensity on fMRI of the contralesional side is associated with slower, reduced recovery in human studies, indicating that this excitability could suppress improvements in motor ability, therefore suppressing positive neuroplastic changes in the motor cortex, ultimately reinforcing learned misuse. The application of TMS in these cases is centred around suppressing the inhibitory contralesional signalling to allow for productive motor system reorganisation to occur following a stroke.

Hemiparesis and hemiplegia are common manifestations of stroke, occurring in over 50% of cases. Axons of upper motor neurons primarily descend from the primary motor cortex, supplementary motor area, and the premotor cortex, and together contribute approximately 60% of upper motor neurons within the descending corticospinal tract [7]. These areas receive extensive vascular supply from branches of the middle cerebral artery, which have a higher likelihood of being involved in stroke compared to the rest of the anterior and posterior circulation [8]. As such, the anatomical position of middle cerebral artery strokes disconnects areas of the motor cortex from their caudally projecting tracts, either through direct insult to the cortex, to the subcortical descending nerve fibres, or both. Therefore, fibres carrying descending motor inputs, including those to the spinal cord and other subcortical targets, are lost. However, subcortical targets of motor signals maintain connections to both cortices, as well as perilesional areas; therefore, focal motor deficits do not indicate a complete severance of these tracts from the cortex [7]. This permits the possibility of neuroplastic changes to regain lost motor function.

Observed improvements in upper and lower limb hemiplegia vary significantly. Upper limb hemiplegia poses a greater challenge to neurorehabilitation, and full recovery remains exceptionally difficult for most patients to attain, if attainable at all. Lower limb hemiplegia may pose greater limitations on mobility; however, rehabilitative efforts often prove to be more effective in producing functional improvements in this patient population. This may be due to the somatotopic organisation of the motor cortex in control of these areas; the hand requires greater fine motor control and continuously performs intricate movements within three‑dimensional space through control of the intrinsic muscles of the hand, forearm, upper arm, and shoulder. Therefore, a greater portion of the cortex is reserved for upper limb function [9]. Comparatively, much less of the primary motor cortex is responsible for lower limb movements, including those of the foot and the anterior aspects of the upper and lower leg. Again, it is thought that this corresponds to the complexity of movements required of that area. Therefore, vicarious changes in response to lower limb hemiplegia require less use of the maintained functional reserve following a stroke, allowing for faster functional recovery, particularly when aided by physiotherapeutic approaches. The opposite may be true for upper limb hemiplegia; a significant amount of functional reserve must be reorganised to take over the functions of the upper limb M1, therefore the time taken for this to be achieved, as well as the complexity of motor tasks that need to be relearned through neurorehabilitation, leads to slower and often less significant improvements in motor function [6].

Depending on the extent of cerebral tissue damage, varying degrees of reorganisation may occur. For lesions contained within the primary motor cortex (M1), it is thought that redundant perilesional neurons are induced as a substitute for the ischaemic area through somatotopic reorganisation-this is an example of vicariation. Convincing evidence of somatotopic cortical reorganisation has been demonstrated in chronically hemiplegic stroke patients through longitudinal fMRI studies [10]. Functional reorganisation within surviving perilesional regions surrounding infarcts restricted to the primary motor cortex has been observed over the course of recovery. The notion that M1 is capable of spontaneous adaptation in humans is supported by these studies, yielding results comparable to those seen in primate and mouse models. Mouse models suggest that this could be mediated through preservation of the ischaemic area, which acts as a substrate for surviving, albeit redundant nerve fibres to sprout and reinnervate subcortical targets [9]. Both human and animal studies have contributed greatly to the evidence base for adaptive perilesional reorganisation, while also demonstrating its importance in functional rehabilitation. These changes in cortical topology observed through fMRI are shown to be predictive of accelerated motor recovery and better motor function; however, results still differ between studies. Inter‑subject variability with regard to the level of function, the exact location of the lesion within M1, initial rehabilitation trajectory, and intensity of neurorehabilitation therapy must be accounted for to allow sequitur conclusions to be drawn on the mechanisms mediating these changes [10].

In cases of wider neurological damage, there may not be an adequate reserve of local surviving cortical neurons for somatotopic reorganisation. A proposed mechanism for overcoming this is the employment of functionally similar regions of the cortex, which are not anatomically in direct connection with the affected area, namely, the supplementary motor area (SMA), which has been the focus of much research [11]. The SMA is located medially within Brodmann area six, bounded posteriorly by the precentral gyrus (M1). The functional importance of this area has long remained ambiguous since its discovery in 1951, yet studies have found significant links between the SMA and planning/sequencing movements, bimanual motor actions, as well as direct, self‑initiated motor outputs. More recent studies also put forward the idea of two subdivisions within the SMA; the pre‑SMA sits rostrally and is involved in more abstract and wider cognitive functions such as spatial processing, timing and working memory, while the SMA proper sits caudally in direct connection with M1, with connections projecting to both M1 and the premotor cortex to aid in the execution of movements, as well as to the spinal cord, creating an alternative pathway for self‑initiated movements [11]. In hemiplegic stroke patients with significant functional improvement, reduced excitability is often observed in the corticospinal tract using motor evoked potentials, suggesting that such an alternative path may have contributed to recovery [12]. Furthermore, a study has shown that the absence of fMRI activation in the SMA proper was predictive of slow and poorer recovery, whereas greater activation was correlated with better, faster recovery [10]. In these patients, the SMA may have played a crucial role in recovery through utilising alternative motor output pathways and forming new neural networks to bypass corticospinal disruption.

Bihemispheric reorganisation of surviving neural networks following injury to the cortex has been observed in human models. Functional MRI studies have displayed an expansion of sensorimotor representations to both hemispheres following stroke. While this pattern has been observed frequently in chronic stroke patients, it is unclear how remote regions of the cortex contribute to motor recovery. Numerous studies have shown that ipsilateral reorganisation is a better predictor of improved motor function, while bihemispheric reorganisation is often found among those who maintain significant motor deficits. In addition, those with significant bihemispheric reorganisation who regain motor function often display an evolution of cortical activation in fMRI studies, in which signalling transfers to the ipsilesional side. Meanwhile, poorly recovered patients continue to show activation in the contralesional hemisphere [13]. Nevertheless, studies fail to draw a causal link between bihemispheric reorganisation and poor motor function - does it inhibit effectual motor reorganisation by producing an interhemispheric imbalance? Or is it simply an effect of irremediable functional motor loss? In contrast, there is evidence to suggest that bihemispheric reorganisation may play a key positive role in motor recovery. In patients who have specifically experienced striatocapsular strokes, approximately 90% of the ipsilesional corticospinal fibres are lost [13]. Contralesional fMRI activation has been found in patients in this cohort who show significant motor improvement, suggesting that reorganisation to the contralateral hemisphere may be effective under these circumstances. As ipsilesional corticospinal fibres (both functional and redundant) are widely affected, limitations in ipsilesional plasticity and input may permit greater contralesional input. However, this could be a misinterpretation, as contralesional activation may also be indicative of an upregulation of activity in a pre‑existing motor network, rather than being the consequence of newly formed neural networks. There is much uncertainty regarding the role of bihemispheric reorganisation; therefore, its role in rehabilitative TMS has been the focus of much research.

rTMS

Modern literature suggests that rTMS facilitates neuroplastic changes through inducing long‑term potentiation (LTP) and long‑term depression (LTD). Both mechanisms demonstrate how changes in synaptic connectivity may be altered by experience. Long‑term potentiation is thought to occur following transient high‑intensity depolarisation of postsynaptic neurons. Conversely, LTD arises from prolonged low‑intensity stimulation or continual activation following successful depolarisation. Both mechanisms are the result of α‑amino‑3‑hydroxy‑5‑methyl‑4‑isoxazolepropionic acid (AMPA) receptor regulation through an N‑methyl‑D‑aspartate (NMDA) receptor‑dependent mechanism. In LTP, rapid postsynaptic activation of AMPA receptors by glutamate facilitates the influx of Ca²⁺ via the NMDA receptor, which initiates a Ca²⁺/calmodulin‑dependent protein kinase II (CaMKII)‑dependent cascade to upregulate the AMPA receptor. Ca²⁺ entry also facilitates phosphorylation of AMPA via CaMKII, increasing its single-channel conductance. This leads to greater sensitivity and the number of these receptors in the membrane, and thus greater sensitivity of the postsynaptic neuron, increasing the frequency of successful action potentials [14]. Repetitive high‑frequency TMS of ≥5 Hz has been extensively connected to the induction of LTP. An increasing number of studies demonstrate that the amplitude of motor evoked potentials (MEPs) induced by single‑burst TMS of the motor cortex is increased following rTMS (≥5 Hz). Significant increases in amplitude have been observed after 900 consecutive pulses, suggesting that rTMS is directly influencing neural plasticity and strengthening corticospinal connectivity [15]. In hemiplegic stroke patients, using rTMS to enhance the sensitivity of postsynaptic neurons in the motor cortex could aid as an adjuvant rehabilitative intervention in those with limited damage to the ipsilesional motor cortex. In LTD, there is insufficient AMPA‑facilitated depolarisation to liberate Mg²⁺ from a significant proportion of postsynaptic NMDA receptors; therefore, the influx of Ca²⁺ via NMDA is reduced, and greater time is taken for the threshold potential to be achieved. The cytoplasmic calcium concentration is too low to induce the same CaMKII‑dependent cascade present in LTP, which in turn promotes downregulation of AMPA receptors, and thus lower sensitivity and reduced response to glutamate signalling. It is thought that LTD can be induced by low‑frequency rTMS of ≤1 Hz, and numerous studies show that low‑frequency rTMS suppresses the amplitude of MEPs induced by single‑pulse TMS. It can be argued that there is good evidence to suggest that 1 Hz rTMS produces some reduction in corticospinal excitability and, therefore, its use in suppressing detrimental hyperexcitation, such as in interhemispheric competition, could play a role in stroke rehabilitation [16].

Low-frequency r‑TMS

As discussed, the brain has significant capacity for reorganisation following a stroke. Further, interhemispheric imbalances may impede the capability of the cerebral cortex to undergo these processes. Abnormal interhemispheric inhibition from the unaffected hemisphere to the affected hemisphere is commonly observed in stroke patients. In healthy subjects, low‑frequency 1 Hz rTMS over the non‑performing M1 has been demonstrated to improve function in the ipsilateral hand when performing motor tasks. Positron emission tomography of these subjects revealed increased regional cerebral blood flow in the left premotor cortex during movement of the right hand [17]. Chronic stroke patients with significant motor deficits express increased inhibitory signalling of the contralesional hemisphere to the ipsilesional side during movement preparation; therefore, a proposed treatment strategy is to use low‑frequency, inhibitory TMS to reduce this excitability. Multiple meta‑analyses have concluded that this use of rTMS produces positive effects on finger and hand mobility, which, while modest, are certainly encouraging and support the theory of interhemispheric inhibition playing a key role in motor function suppression [2,18].

However, real‑world functional upper limb use requires adequate control over the proximal muscles, allowing for articulation of the shoulder and elbow, such as to guide the hand within three‑dimensional space. One double‑blind randomised sham‑controlled trial examined the immediate and retained effects of 1 Hz rTMS on the contralesional M1 in stroke patients with upper limb paralysis, at 100% of their resting motor threshold [19]. It concluded that functional deficits of larger joints were not remediated by low‑frequency rTMS, therefore suggesting that this intervention may not be developed to the stage where it can facilitate substantial functional improvement. It is important to keep in mind, however, that low‑frequency rTMS does not deteriorate motor performance of the non‑affected hand - an important note, as if the opposite were true, this could severely impact motor learning and the performance of bimanual tasks.

As mentioned previously, somatotopic differences in regions of M1 responsible for upper and lower limb movement correlate with rehabilitative outcomes for stroke patients. Both high and low‑frequency rTMS can be used to improve the rate of neuroplastic change directly and indirectly, respectively, in both upper and lower limb hemiplegia. However, the extent of plasticity that needs to take place differs due to the size of the regions in control of upper and lower limb movements. While full upper limb functional recovery is not currently a realistic outcome of low‑frequency rTMS‑assisted rehabilitation, there is significant evidence suggesting it does improve clinical motor recovery alongside physical therapy in early stages of recovery (i.e., within two weeks) [20]. Another double‑blind randomised sham‑controlled trial assessed the effects of both low‑ and high‑frequency rTMS on upper limb motor function in stroke patients within the first two weeks of recovery [21]. The primary outcome measure was the Upper Extremity Fugl‑Meyer score evaluated at baseline, after rTMS intervention, and at the three‑month follow‑up, as well as fMRI data recorded at each visit. It found that, when compared to the control (sham) group, clinical motor improvement was significantly greater in those who received inhibitory contralesional rTMS. Furthermore, inhibitory rTMS produced greater ipsilesional M1 activation at three months when compared to baseline. However, these results were far less pronounced than those of the high‑frequency rTMS groups. Functional MRI reports of these patients did reveal that low‑frequency stimulation suppressed contralesional hyperactivity; however, the significance of this when facilitating significant motor improvement is unclear. Where ipsilesional M1 injury is significant, inhibitory rTMS could theoretically provide greater benefit to upper limb movement; however, rebalancing interhemispheric inhibition may not be conducive to significant motor recovery. Future studies should focus on the specific effects of interhemispheric inhibition and test whether modulation of cortical networks with low‑frequency rTMS could be a critical mediator of functional upper limb motor recovery.

Lower limb function may also be modulated using inhibitory rTMS. Walking and balance are frequently impaired in stroke patients and significantly affect quality of life; higher falling cadence, prolonged gait cycles, and gait asymmetry are common manifestations in stroke patients. Low‑frequency rTMS may be a valid aid in the recovery of normal lower limb function. While undergoing physical and occupational therapy alone, most stroke patients obtain a varying degree of motor improvement; however, most maintain pathological gait features, such as foot drop, hyperextension at the knee, and external hip rotation to compensate for a lack of balance and motor control. When analysing gait performance in stroke patients undergoing inhibitory rTMS of the contralesional M1, two studies [22,23] found that low‑frequency stimulation combined with task‑oriented physical therapy produces significant improvements in motor control and walking ability, compared to task‑oriented therapy alone. Furthermore, one of these studies explored the effect of inhibitory rTMS on walking cadence and speed and found that 15 minutes of 1 Hz rTMS for five consecutive days facilitated greater motor control, a reduction in pathological gait patterns, and reduced lower limb spasticity when compared to pre‑intervention and sham stimulation. The results of each study supported the idea that low‑frequency rTMS had a positive influence on enhancing motor function, especially in those who experienced lower limb spasticity, leading to an overall improvement in the capability of performing activities of daily living. It could be proposed that, for lower limb dysfunction, low‑frequency rTMS should be considered as a primary adjuvant therapeutic technique alongside physical therapy, given there are no contraindications.

High-frequency rTMS

An alternative approach is to stimulate the ipsilesional hemisphere with higher‑frequency rTMS. If vicariation has occurred on the affected side, this could help strengthen new neural networks through inducing plastic changes by encouraging LTP. Further, high‑frequency trains of rTMS may augment normal activity within the targeted area and help to suppress interhemispheric inhibition from the contralesional side. There is a significant evidence base to suggest that this is an effective modality in aiding short‑ and long‑term recovery. In upper limb hemiplegia, high‑frequency rTMS may also increase upper limb function more successfully than physiotherapy alone. Vabalaite et al. [24] carried out a systematic review of four randomised controlled trials exploring the application of rTMS in upper extremity paresis following stroke. Again, the primary outcome measure in most of these studies was the Fugl‑Meyer Assessment, used to assess both the intervention and control groups. Each study used 10 Hz rTMS over the ipsilesional M1, and all four studies conveyed better results relating to hand motor function in the rTMS group compared to the control. Across the four studies, six patients experienced headaches, supposedly induced by rTMS, and four of these patients could not tolerate further sessions and therefore were removed from the trial. No other harm or significant adverse events were reported by any of the trials. The overall result from the review was that high‑frequency rTMS may increase impaired upper limb function when compared to sham stimulation. However, the reliability and reproducibility of the results of these trials may be impacted by their small sample sizes, with fewer than twenty patients in the intervention group of three of these trials. This, however, is a common theme for rTMS randomised controlled trials, and future studies with larger sample sizes will be necessary to better estimate the efficacy and safety of high‑frequency rTMS in upper limb motor function recovery.

The application of high‑frequency rTMS in the lower limb motor area has not been thoroughly investigated, especially in the acute stages of stroke. Approximately 20-30% of pyramidal tract input is ipsilateral, and this primarily controls the trunk and lower limbs [7]. When hemiparesis is induced by stroke, the trunk and proximal limbs on the non‑paretic side are also impacted, although not to the degree of the paretic side. Functional MRI studies show that increased activity in bilateral motor areas correlates with improved function, so it may be hypothesized that high‑frequency rTMS of both leg motor areas would be beneficial in improving the function of both the paretic leg and body trunk. One study [23] observed how high‑frequency rTMS over both the ipsilesional and contralesional M1 leg motor area influenced functional recovery in the early phase (10.9 ± 6.6 days after onset) of stroke. In this randomised sham‑controlled trial, rTMS was applied bilaterally using a double cone coil in 10 Hz trains for 10 seconds with 50 Hz inter‑train intervals (1000 pulses in total per session), alongside a standard physiotherapy regimen for five days. The Brunnstrom Recovery Stages and Ability for Basic Movement Revised scales were used to assess baseline and post‑intervention function. All patients showed significant improvements in both metrics, and no patients reported any pathological symptoms or neurological deterioration. However, those who received high‑frequency rTMS scored significantly higher in both metrics after five days compared to the sham stimulation group, suggesting that this intervention is safe and feasible for motor function recovery in the paretic leg and trunk in the early phases of stroke.

Changes in propulsive forces and overall walking speed have been observed in chronic hemiplegic stroke patients undergoing supplementary high‑frequency rTMS. Another trial investigated how high‑frequency rTMS would impact walking function in chronic stroke patients. The study was randomised, double‑blind, cross‑over in design, and all patients were scheduled to receive both sessions of high‑frequency rTMS and sham stimulation. These patients were able to walk a minimum of 10 m without assistance; however, they all still retained significant gait disturbances. Walking velocity was used as the primary outcome measure, as this has been reported to correlate with other sophisticated methods of measuring motor function improvement. Following 20 minutes of high‑frequency rTMS (10 Hz trains for 10 seconds with 50 Hz inter‑train intervals for 20 minutes - a total of 2000 pulses), walking velocity was measured immediately, at 10 minutes, and 20 minutes in both the sham and intervention groups, then the groups were swapped, and the study was repeated 24 hours later. When instructed to walk as briskly as possible, it was found that in both groups, after 20 minutes of stimulation, significant differences in walking speed were observed between the treatment and sham groups. The high‑frequency rTMS group showed significantly higher walking velocity compared to the sham group at all three intervals. The increase in walking velocity immediately post‑intervention was maintained until 20 minutes after the stimulation. In the sham group, there was no significant increase immediately after stimulation, but there was a significant difference at 10 minutes and 20 minutes after stimulation compared to before stimulation. No carry‑over of effects on walking function was observed after 24 hours, which indicates that this study was an effective measure of the immediate effects of high‑frequency rTMS. However, it fails to show that this intervention provides a long‑term therapeutic benefit. Frequent application of rTMS over several days in chronic stroke patients could lead to retained improvements in walking ability, and future research should aim to explore the feasibility of high‑frequency rTMS as a long‑term therapeutic intervention [25].

Applying rTMS in stroke recovery

It is vital to note that rTMS alone is unable to promote significant functional improvement and independence [26]. Repetitive TMS should function as a catalyst for standard rehabilitative approaches, and not a substitute for them. All members of the multidisciplinary team involved with stroke patients are vital for the rehabilitation process: physiotherapy not only encourages the plastic changes observed following stroke, but also maintains the muscle bulk which the motor cortex controls. Long‑term limb misuse promotes muscular atrophy, which in turn impedes functional recovery. Therefore, maintaining the muscles necessary to perform specific movements is arguably as important as the reorganisational processes involved in motor retraining. Occupational therapy is also vital in the transition between regaining motor function and performing meaningful actions, which patients will recognise as indicators of significant recovery. While rTMS is not beneficial in isolation, its application alongside other therapeutic methods is believed to strengthen the neural networks formed through motor retraining and neural plasticity, which ultimately will produce faster recovery times and overall reduce retained motor impairments in stroke patients. The only way in which rTMS may be effectively applied as a standard rehabilitative procedure is through the work of an effective and attentive multidisciplinary team capable of assessing a patient's therapeutic needs and deciding if they are an appropriate candidate for rTMS therapy [27].

Motor improvements following stroke tend to adhere to a nonlinear logarithmic pattern, with the majority of motor improvement occurring within the first three months [28]. However, recovery is not simply limited to the acute phase; the brain may still undergo plastic changes far beyond the acute and subacute phases, as improvements in upper and lower limb paralysis have been observed years after a stroke. Nevertheless, stroke recovery patterns remain heterogeneous with regard to functional outcomes. Patients with mild to moderate upper extremity hemiplegia in the acute phase of stroke have a favourable outlook for functional recovery, with 71% of these patients achieving at least some dexterity within six months after stroke. In contrast, patients with severe unilateral weakness have a poor prognosis, with approximately 60% failing to regain dexterity in the same timeframe [28]. Only a small percentage of patients who initially experienced complete paralysis are able to achieve functional use of their arm. In the acute phases, contralesional low‑frequency rTMS could provide substantial benefit to patients with significant interhemispheric inhibition impeding on retained motor function and local reorganisation. In later stages, if significant functional improvement has been attained, high‑frequency rTMS could strengthen the existing reorganisation to reduce gait disturbances and improve retrained motor processes. Beyond three months, retained motor dysfunction due to stroke is generally considered chronic pathology. Neurological recovery often reaches a plateau, such that rehabilitative responses usually no longer reap major positive benefits. However, much of the research base for rTMS has targeted patients beyond six months of onset, and widely positive outcomes suggest that motor function changes can be facilitated by rTMS. This allows for further beneficial continuation of rehabilitation for stroke patients who retain functional deficits [28].

Different patient populations have shown varied responses to rTMS. Understanding how specific characteristics may predispose to better outcomes would be helpful to match patients to specific methods of rTMS, therefore enhancing its benefit. It is currently unknown which characteristics may determine individual responses to rTMS. One study [29] found a trend between stroke site and movement kinematics following ipsilesional 10 Hz rTMS, with patients who experienced subcortical infarcts showing greater improvements following intervention, compared to those with cortical infarcts. This could be explained by the preservation of interhemispheric pathways, therefore limiting interhemispheric inhibition in the subcortical group, as interhemispheric inhibition is generally decreased in subcortical strokes relative to cortical strokes. Overall size of the ischaemic region may also be a good indicator, as this will influence the degree of vicariation and the amount of interhemispheric inhibition taking place. This is reinforced by the findings of two sham‑controlled trials that show high‑frequency rTMS to be less effective in severe chronic hemiparesis, while inhibitory rTMS precipitates superior outcomes when compared to sham stimulation [25]. From this, it can be suggested that even in severe chronic cases with significant loss of ipsilesional motor cortex input, flexible use of rTMS can produce benefits.

Wider benefits of rTMS therapy in stroke

The incidence of stroke in the UK is predicted to rise markedly in the coming years. One in six people in England will have a stroke during their lifetime, 30% of whom will go on to experience another event. Stroke remains a leading cause of death and disability; however, over the past 15 years, stroke mortality has decreased significantly, likely due to earlier and more effective intervention in the acute stage, alongside greater awareness of the health implications of poor diet and smoking [1]. This, in turn, means that more people are affected by the disabling aspects of stroke, the most common of which are motor impairments (alongside speech and language pathology and cognitive deficits). Dependency in activities of daily living persists in around 35% throughout the first year following stroke [30]. Early rehabilitation is key to reducing the burden of stroke‑related disability, and rTMS aims to increase both the rate and quality of functional improvement throughout the recovery process.

The Barthel index is an ordinal scale used to measure performance in activities of daily living for stroke patients. It assesses a range of activities, including feeding and bathing, as well as functional mobility. Current research is consistently positive regarding the impact of rTMS on a patient's Barthel index following stroke. One study found a significant increase in the Barthel index after four weeks of inhibitory (1 Hz) rTMS for ten 30‑minute sessions over four weeks. In this study, a cohort of 42 chronic stroke patients with upper limb hemiplegia (3-12 months post stroke) received either rTMS coupled with motor imagery therapy, another non‑invasive neuromodulation technique, or motor imagery therapy alone [31]. Despite the limitations of this study, such as a small sample size and limited follow‑up time, it concluded that rTMS, alongside motor imagery in chronic stroke patients, obtained better improvements in the Barthel index than the non‑rTMS group. Similarly, another study observed how a single session of high‑frequency (5 Hz) rTMS improved the Barthel index in subacute, subcortical middle cerebral artery stroke patients with varying motor pathology, when compared to sham stimulation [29]. After only two days of stimulation, it was found that patients had higher Barthel index scores when compared to the sham group. Scores remained improved for one month; however, when measured at three, six, and 12 months, there was no improvement when compared to the control group. Fugl‑Meyer scores remained consistently higher in the rTMS group. Repetitive rTMS may facilitate greater ability to perform activities of daily living in stroke patients; however, the longevity of these effects appears limited [31].

Reducing chronic disability through novel interventions in stroke rehabilitation also has wider beneficial economic impacts. Stroke results in an estimated burden of several billion pounds a year to health and care services in the UK, with significant costs arising from outpatient and community care costs and from informal care costs. These costs are exacerbated by long‑term disability and greater dependency with activities of daily living, as well as the cost of physiotherapy and occupational therapy provision. Lower Barthel index scores are associated with lower overall care costs, likely due to lower need for continuous supervision and around‑the‑clock care. Improvements in the Barthel index are less profound in the chronic stage of stroke disability, and traditional therapeutic approaches often do not provide any further benefit. As discussed, current literature correlating rTMS with changes in the Barthel index in both the acute and chronic stages of recovery has been consistently positive, although the permanence of these effects remains in question [31].

Learned misuse of affected limbs, particularly of the lower limbs, may potentiate low physical activity, a major modifiable factor contributing to stroke mortality. In chronic cases of lower motor disability (i.e., more than three months), rTMS has been shown to be effective in improving gait performance in stroke patients, which subsequently improves overall motor performance and walking capability. Improving confidence in walking not only decreases dependency for activities of daily living but may also encourage engagement in physical activity and improve the efficacy of physiotherapy. Regular low‑intensity exercise, such as walking, has been shown to have a protective effect on stroke recurrence, with every hour performed per week reducing recurrence risk by 8.8% [32]. Chronic stroke patients are particularly susceptible to learned misuse, as therapeutic efforts may be focused on functionality, rather than regaining lost motor function. Therefore, helping those with lower motor dysfunction in the chronic phase of stroke recovery to regain and improve their motor ability through rTMS could have wider effects on mortality by reducing recurrence in the long term [22].

At the time of this review being written, rTMS is not offered as a standard rehabilitative method for functional recovery in stroke patients. The National Institute for Health and Care Excellence does not currently recommend rTMS for post‑stroke motor recovery; however, it currently supports its application in numerous psychiatric disorders. The rationale behind this stems from a 2013 Cochrane review of 19 randomised controlled trials, which concluded that rTMS was not associated with a significant improvement in activities of daily living [33]. As discussed, much of the current research has not yielded results suggestive of rTMS facilitating full functional recovery. Most of the trials mentioned in this writing, as well as many of those forming much of the evidence base for rTMS, also suffer from significant drawbacks, which therefore limit the conclusions drawn from this review. All trials reviewed reported small sample sizes, ranging between 12 and 60 participants. Due to its low statistical power, the current evidence base is susceptible to type II errors, and the results should be interpreted with caution. These trials may not have the power to accurately discern the efficacy of rTMS against the sham groups, echoing the need for larger-scale randomised controlled trials to verify these results. Also, all but one of the trials evaluated the outcome at (or near) the end of the treatment period, which did not allow for the assessment of the long‑term effects of rTMS therapy. A change to the national guidelines would require both larger randomised controlled trials and greater consistency between the methodology of these trials to inform specific and instructive suggestions regarding rehabilitative care [33].

## Conclusions

Repetitive transcranial magnetic stimulation successfully modulates motor function in stroke patients. Efforts in applying rTMS as a therapeutic measure in stroke have created an extensive knowledge base on the subject; however, more research needs to be done to justify its widespread use. The success of these efforts has also supported our current understanding of how the brain reorganises following a stroke. Further studies should focus on producing long-term functional benefits through rTMS, standardising dosage parameters, and showing consistent and repeatable results through larger randomised control trials. Nevertheless, rTMS is a promising therapeutic strategy, and when applied as an adjuvant rehabilitative technique, could lead to significant enhancements in motor recovery. 
